# Michel Brémont (1953–2020)

**DOI:** 10.1186/s13567-020-00878-7

**Published:** 2020-12-30

**Authors:** Stéphane Biacchesi, Sabine Riffault, Thierry Pineau, Muriel Vayssier-Taussat, the Staff and Associate Editors from Veterinary Research, Vincent Béringue

**Affiliations:** 1 Université Paris-Saclay, INRAE, UVSQ, Jouy-en-Josas, VIM France; 2 Université Paris-Saclay, INRAE, Jouy-en-Josas, France; 3 Animal Health Division, INRAE, Tours, France

Dear colleagues,
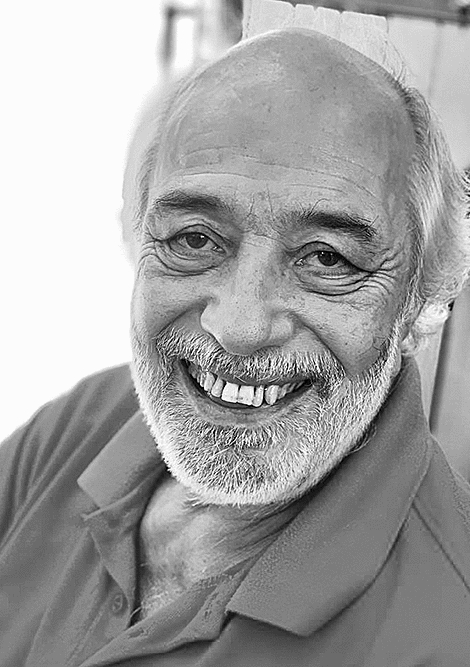


We share with you the painful news of the death of Michel Brémont. Michel has succumbed to cancer on the night of November 10^th^, 2020. He was 67 years old.

Michel was a researcher recognized for the quality and extent of his innovative work on infectious diseases of fish. He is known for his commitment to establish reverse genetics systems on fish RNA viruses and to develop original virus vectors and vaccine platforms. With his characteristic determination, he also invested in the creation of understanding and knowledge in the development of preventative solutions for fish farm species but also for mammals. His notoriety was international and we know that the news of his passing widely affects the community of his collaborators and colleagues in many countries.

Among the Molecular Immunology and Virology (VIM) unit at INRAE, Michel was an inspiring leader and a flawless supporter for his close associates. He had a sense of the collective and a concern for others.

He passed on his expertise, his values, and his tenacity to young scientists as the former leader of the Fish Molecular Virology team. Beyond the VIM unit, for his colleagues in the INRAE Animal Health Division, he remains a “character”, the symbolic figure of researcher honesty, passion, stood up for his convictions with vigor (and sometimes of bad mood) who has guided many of us in their careers.

With his extensive and eclectic scientific knowledge, his determination to lead his projects, his motivation to give and to shape and his candid way of speaking, Michel reached success with each mission that makes up the range of jobs of Researcher.

Among his exemplary involvement for the scientific community, the responsibility of Editor-In-Chief of the journal “Veterinary Research”, assumed a unique dimension. With clear-sighted strategy, Michel learned to guide the journal to be a beacon of the international infectious disease community in his move to go to “open access”, the change of his economic model expanded his renown and his readership. This important community of readers is totally in his debt.

On this sad day of the news of the passing of Michel Brémont, our compassionate and kind thoughts go to his loved ones, particularly to his children: Adrien, Antoine, Camille, and Sarah.

We keep the faithful and inspiring memory of Michel as a man of passion and huge generosity, a lively rare power of conviction always tinted with humor. The beautiful picture that we attached to this message, which was transmitted to us by his children, perfectly illustrates his generous and warm personality.

